# Does parkland influence walking? The relationship between area of parkland and walking trips in Melbourne, Australia

**DOI:** 10.1186/1479-5868-9-115

**Published:** 2012-09-19

**Authors:** Tania L King, Lukar E Thornton, Rebecca J Bentley, Anne M Kavanagh

**Affiliations:** 1 Centre for Women’s Health, Gender and Society, Melbourne School of Population Health, University of Melbourne, Melbourne, Australia; 2 Centre for Physical Activity and Nutrition Research, School of Exercise and Nutrition Sciences, Deakin University, Victoria, Australia

**Keywords:** Walking frequency, Park area, Park proximity, GIS, Multilevel

## Abstract

**Background:**

Using two different measures of park area, at three buffer distances, we sought to investigate the ways in which park area and proximity to parks, are related to the frequency of walking (for all purposes) in Australian adults. Little previous research has been conducted in this area, and results of existing research have been mixed.

**Methods:**

Residents of 50 urban areas in metropolitan Melbourne, Australia completed a physical activity survey (n = 2305). Respondents reported how often they walked for ≥10 minutes in the previous month. Walking frequency was dichotomised to ‘less than weekly’ (less than 1/week) and ‘at least weekly’ (1/week or more). Using Geographic Information Systems, Euclidean buffers were created around each respondent’s home at three distances: 400metres (m), 800 m and 1200 m. Total area of parkland in each person’s buffer was calculated for the three buffers. Additionally, total area of ‘larger parks’, (park space ≥ park with Australian Rules Football oval (17,862 m^2^)), was calculated for each set of buffers. Area of park was categorised into tertiles for area of *all* parks, and area of *larger* parks (the lowest tertile was used as the reference category). Multilevel logistic regression, with individuals nested within areas, was used to estimate the effect of area of parkland on walking frequency.

**Results:**

No statistically significant associations were found between walking frequency and park area (total and large parks) within 400 m of respondent’s homes. For total park area within 800 m, the odds of walking at least weekly were lower for those in the mid (OR 0.65, 95% CI 0.46-0.91) and highest (OR 0.65, 95% CI 0.44-0.95) tertile of park area compared to those living in areas with the least amount of park area. Similar results were observed for total park area in the 1200 m buffers. When only larger parks were investigated, again more frequent walking was less likely when respondents had access to a greater amount of park area.

**Conclusions:**

In this study we found that more park area in residential environments reduced the odds of walking more frequently. Other area characteristics such as street connectivity and destinations may underlie these associations by negatively correlating with park area.

## Background

There is growing evidence of the importance of parks in promoting health and wellbeing [1,2]. Access to parks has been associated with many health benefits including higher self-rated health [3], lower mortality from all causes [4], reduced stress and a lower likelihood of obesity [5], reduced rates of respiratory and cardiovascular disease for males [6], and among children, a higher likelihood of being a healthy weight [7].

Walking is the most common form of physical activity in both Australia [8,9] and elsewhere such as the United States [10,11]. It is clear that activities of light to moderate intensity, such as walking, can deliver significant health benefits [12], [13]. Despite this, and the fact that it is easy for most people to engage in, determinants of walking remain under-investigated.

Several studies have examined the role of parks in promoting higher levels of physical activity [14-19], however the relationship is not straightforward [20]: some studies have found no relationship between park access and physical activity levels [15,21-23], others have found positive associations [14,17,24,25]. Mixed results have also been reported [26,27].

Literature describing the role of parks in promoting walking is sparse [28], particularly in an Australian context. We are aware of some studies that have examined the relationship between parkland accessibility (measured either in terms of area or proximity) and levels of walking [23,26,29-36]. Results of these studies have been equivocal.

The growing application of Geographic Information Systems (GIS) has facilitated more sophisticated measures of access to parks, with methods now enabling researchers to calculate estimates that are specific to an individual’s household address [37]. Previously many researchers have relied on area measures aggregated to an administrative unit such as post code to investigate the links between parks and walking, however there is increasing use of buffers (buffer areas) or distance measures specific to each individual respondent [29,30,32,35,38].

It is important not to make assumptions about the distance at which exposures have an effect. While most studies examining the relationship between walking and parks have included some form of spatial referent, few have looked at multiple specific distances. For this reason, we used three different buffer distances to test the effect. Additionally, most other studies have included all parks in analysis, without considering that the size of parks may matter. Further, it is important to use the most appropriate research design to consider how people’s walking might be influenced by their local environments. Multilevel study designs and analyses allow simultaneous consideration of characteristics of geographic areas and the people who reside in them. It is possible that inadequately accounting for some of these factors may explain some of the previous mixed findings on the association between parkland and walking [20].

We theorised that parks could encourage local walking by improving neighbourhood aesthetics, by offering a destination to walk to and in, and by offering a short-cut to other destinations. Using data from a multilevel study conducted in Melbourne, Australia, in 50 small areas among 2305 respondents, this paper advances previous research by examining the relationship between frequency of walking (for all purposes) and distance to, and size of parkland. While there is evidence that the determinants of walking vary by walking purpose [20], several other studies provide a precedent for the use of an overall walking measure [26,39,40]. We use two different measures of park area (park area of all parks and park area of larger parks) at three different buffer distances (400 m, 800 m, 1200 m), and measure access to parkland from people’s homes*.*

## Methods

### Sampling design

Data from the 2003 VicLANES (Victorian Lifestyle and Neighbourhood Environment Study) project was used. VicLANES was a large, cross-sectional and multilevel study, and its methodology has been reported elsewhere [41,42]. Briefly, the study was conducted across the 21 innermost local government areas (LGAs) in Melbourne, Australia. Census collection districts (known as CCDs, these are the smallest geographic unit of measurement used by the Australian Bureau of Statistics (ABS) in the collection of census data) in all of these LGAs were identified, and ranked according to the proportion of households with a weekly pre-tax income of less than $400/week. This ranking was then stratified into septiles, and a random sample of CCDs was selected from the top (17), middle (16) and bottom (17) septile with a total of 50 CCDs and 19 LGAs (there were between one and five CCDs in each of the sampled LGAs). Within these CCDs, surveys about physical activity were sent to 4,005 residents over the age of 18 years, who were randomly selected from the electoral role (voting is compulsory for all Australians over the age of 18). A 58.7% valid completion rate was achieved, with 2,349 residents returning a valid survey about their physical activity behaviour.

### Outcome measures: Frequency of walking

A closed response question asked respondents about their frequency of walking in the previous month. Specifically, respondents were asked *“How often in the LAST MONTH did you WALK, for 10 minutes or more. Think of any time you walked for recreation or exercise, or to get to or from a specific place (*e.g. *to work, to the post office or shop, to a friend’s house etc.)”*. Respondents were required to tick one of six response categories: never; about once or twice, about once a week, about 2-3 times a week, about 4-5 times a week, every day. Those respondents who ticked “never” followed a skip, and were asked no further questions about walking. Based on the distribution of responses, two response categories were created from this question: less than twice a month (referred to as ‘less than weekly’ walkers); once a week or more (referred to as ‘at least weekly’ walkers).

### Park Area measures

The Public Open Space Data for the Greater Melbourne Area was obtained from the Australian Research Centre in Urban Ecology (ARCUE). The Open Space dataset contains areas identifiable as open green space. This dataset contains comprehensive information on open space in Melbourne, and classifies all open space by type (for example, educational/institutional, cemetery, reserve/park, sporting/recreational, military), and whether it is publicly accessible. Only fully accessible, existing open space (herein referred to as parks) were selected from the dataset (restricted and proposed parks were deleted). School playing areas/fields were not included in the analysis, because public access outside school days was not guaranteed (most school fields in Melbourne Australia are closed after school hours). Golf courses were similarly excluded, because their access is restricted to those playing golf.

There are many differences between parks. While we were unable to explore the qualitative aspects of parks, we were able, and interested in exploring the effect of park area/size. It is possible that parks need to be a certain size to encourage walking. Indeed we felt that house block sized parks, large botanical gardens, large natural parkland, small playgrounds; large parks with multiple football ovals could differentially encourage walking.

Park area was modelled in two ways: firstly as total area of all parks; and secondly, based on a size restriction. With no guidance in the literature to assist in the selection of size, it was reasoned that an Australian Rules football oval is a space that satisfies several criteria:

1. It is of a size that could feasibly encourage walking within it;

2. It is a size large enough to provide a destination that people may travel to, to engage in other forms of physical activity;

3. It is meaningful in an Australian context because it is a type of park commonly found in many neighbourhoods.

The smallest park that had a football field was an area of 17,862 sq. metres. This equates to 4.37 acres. All parks with an area of 17,862 sq. metres or more were then selected (a total of 399, or 31.4% of the total open space dataset). For the purposes of this paper, parks equal to or larger than a football field are referred to as ‘larger parks’.

The distribution of total and ‘larger’ park areas were not normal, and were therefore divided into tertiles, and fitted as categorical predictors with the lowest tertile as the reference.

### Buffers

While both network and Euclidean buffers can be used to measure access to features of the built environment [43], pedestrian travel to playgrounds is thought to be typified by Euclidean distances [44]. By their nature, network buffers are created along roads and paths. If a road runs alongside a park, then the road will form the border of the network buffer, and the park will not be counted. Furthermore, most parks are porous, or do not have barriers, so it makes little sense to measure them according to a road network. Hewko and colleagues [44] argue that pedestrians travelling to parks and playgrounds navigate their way along formal networked pathways such as footpaths, as well as informal ‘short cuts’ and walking paths.

Euclidean buffers of three different scales were created in this analysis: 400 m, 800 m and 1200 m. Although a range of different distances have been used to define walkable distances, there are several strong precedents for the use of 400 m [45-49], and 800 m [17,22]. It is argued that 400 m is the distance that people will walk to, rather than drive [45,46], and approximately equates to a five-minute walk. We chose to use 800 m and 1200 m buffers as they represent the distance that the average person could walk in 10 minutes and 15 minutes respectively. We felt that it was important to use different buffer distances, as they may enable us to capture the different ways that people use parks, and the different ways that parks might influence walking. For example closer parks may encourage more incidental or transport related trips, or trips to the playground, whereas parks further away may act as a destination for longer, less frequent, recreational walks.

Park area was clipped so that only park area inside the Euclidean buffer was included. Table 1 presents the average area of parkland in each tertile, for each of the buffer distances. The fact that the mean park area of the larger parks within a 400 m buffer is smaller than the cut point for larger parks (17862 m^2^) is a consequence of the fact that this method only bisected and encapsulated a fraction of some larger parks.

**Table 1:** Park area by tertile

		**Park tertile**		
		**Tertile 1**	**Tertile 2**	**Tertile 3**
**Park area**	**Buffer**	**Mean (SD) Metres**^**2**^	**Mean (SD) Metres**^**2**^	**Mean (SD) Metres**^**2**^
All Parks	400	5832 (5713)	35748 (12011)	101547 (47327)
	800	77767 (26355)	165841 (31146)	426559 (198384)
	1200	214745 (64526)	429506 (75993)	917064 (327055)
Larger Parks	400	1078 (2075)	26708 (10768)	93345 (49315)
	800	52371 (24207)	138940 (29397)	398308 (203500)
	1200	158164 (60087)	375865 (74450)	852487 (336365)

### Confounders

We included the following variables in the models as confounders because they are likely to be related to walking frequency and the amount of park area close to home: age; sex; country of birth; household type; education; dominant household occupation; disability; and area-level disadvantage.

In relation to occupation, respondents were asked to report their current occupation, as well as their partners. These responses were coded into the ABS’ Australian Standard Classification of Occupations (ASCO) [50], a measure which groups occupations requiring similar levels of education, knowledge, responsibility, on the job training and experience. Responses were then recoded into four categories: professionals (managers, administrators, professionals and para-professionals); white-collar employees (clerks, salespersons and personal service workers); and blue-collar employees (tradespersons, machine operator, drivers, labourers and related workers). The fourth category ‘not working’ was created for those who were retired, studying, unemployed, not looking for work, or unable to work.

With respect to disability, respondents were asked how much they agreed with a statement about whether they had an injury or disability that prevented them from exercising more than they currently do (“I don’t exercise more than I currently do, because I have an injury or disability”). From responses to a five-point likert scale, a binary variable was created for this predictor: “yes” (included those who ticked “strongly agree” or “agree” in response to the statement), “no” (included those who ticked “strongly disagree”, “disagree”, “neither agree nor disagree” to the statement).

Area-level disadvantage was used to define the sample frame (described in the Sampling Design section). The proportion of households with an income of less than $400/week were stratified into septiles, and 50 CCDs were drawn from the top, middle and bottom septile. These three septiles were used in analysis and defined as most advantaged, mid disadvantaged and most disadvantaged strata.

### Statistical analyses

#### Descriptive analyses

The associations between walking frequency and age, sex, country of birth, household type, education, dominant occupation, disability/injury and area disadvantage were compared using a *χ*^2^ test of proportions.

#### Regression analyses

To estimate the associations between park area and frequency of walking, multilevel logistic regression analyses were conducted. Walking frequency was modelled as a binary outcome – less than once a week, once a week or more. As the reference group was ‘less than weekly’, the coefficients estimated in these models indicate the likelihood of moving into the higher category of walking. Multilevel analysis is ideally suited to the analysis of clustered or hierarchical data such as this, as it partitions the variance across levels. In this analysis, we modelled areas as random effects at level 2 and park area as a fixed effect at level 1. Two set of models were fitted:

1. Total park area (with the lowest tertile as the reference category) and frequency of walking, adjusted for potential confounders.

2. Area of large parks (with the lowest tertile as the reference category) and frequency of walking, adjusted for potential confounders.

Odds ratios and 95% confidence intervals are reported for the effect estimates. All analyses were conducted in Stata SE 10.0. A 5% significance level was used for all statistical tests.

## Results

Descriptive statistics of average park area in each buffer, for each tertile are shown in Table 1.

### Socio-demographic characteristics of the sample

The summary table below (Table 2) shows that a higher proportion of women, those with a bachelor degree or higher, professionals and households without children walked most frequently.

**Table 2:** Sociodemographic characteristics by walking

		**Walk frequency**		
	**Total sample n = 2305 (complete case)**	**Less than weekly (walk twice a month or less)**	**At least weekly (walk more than once/week)**	
	**n (%)**	**n (%)**	**n (%)**	p- value*
*Sex*				
Male	1015 (44.0)	228 (50.7)	787 (42.4)	0.002
Female	1290 (56.0)	222 (49.3)	1068 (57.6)	
Missing	0 (0)	0 (0)	0 (0)	
*Country of Birth*				
Australia	1631 (70.8)	284 (63.1)	1347 (72.6)	<0.001
Elsewhere	663 (28.8)	164 (36.4)	499 (26.9)	
Missing	11 (0.5)	2 (0.4)	9 (0.5)	
*Age (years)*				
18-24	182 (8.0)	36 (8.0)	146 (7.9)	0.404
25-34	395 (17.1)	72 (16.0)	323 (17.4)	
35-44	492 (21.3)	113 (25.1)	379 (20.4)	
45-54	495 (21.5)	91 (20.2)	404 (21.8)	
55-64	391 (17.0)	75 (16.7)	316 (17.0)	
Over 65	350 (15.2)	63 (14.0)	287 (15.5)	
Missing	0 (0)	0 (0)	0 (0)	
*Dominant Occupation (household)*				
Professionals	1060 (46.0)	172 (38.2)	888 (47.9)	<0.001
White-collar	352 (15.3)	71 (15.8)	281 (15.2)	
Blue-collar	243 (10.5)	74 (16.4)	169 (9.1)	
Not in labour force	597 (25.9)	117 (26.0)	480 (25.9)	
Missing	53 (2.3)	16 (3.6)	37 (2.0)	
*Education*				
Bachelor degree or higher	719 (31.2)	119 (26.4)	600 (32.4)	0.001
Diploma	257 (11.2)	35 (7.8)	222 (12.0)	
Vocational	431 (18.7)	100 (22.2)	331 (17.8)	
No post school qualifications	831 (36.1)	179 (39.8)	652 (35.2)	
Missing	67 (2.9)	17 (3.8)	50 (2.7)	
*Household type*				
Single adult, no children	397 (17.2)	70 (15.6)	327 (17.6)	<0.001
Single adult, children	133 (5.8)	32 (7.1)	101 (5.4)	
Two or more adults, no children	947 (41.1)	152 (33.8)	795 (42.9)	
Two or more adults, children	778 (33.8)	183 (40.7)	595 (32.1)	
Missing	50 (2.2)	13 (2.9)	37 (2.0)	
*Strata*				
Least disadvantaged	834 (36.2)	167 (37.1)	667 (36.0)	0.691
Mid-disadvantaged	772 (33.5)	143 (31.8)	629 (33.9)	
Most disadvantaged	699 (30.3)	140 (31.1)	559 (30.1)	
Missing	0 (0)	0 (0)	0 (0)	
*Injury or disability*				
Yes	489 (21.2)	114 (25.3)	375 (20.2)	0.03
No	1675 (72.7)	316 (70.2)	1359 (73.3)	
Missing	141 (6.1)	20 (4.4)	121 (6.5)	

*P-values arising from chi-square test for differences in the distribution of predictor and outcome variables across levels of area disadvantage.

### All parks and walking

**Figure 1:**
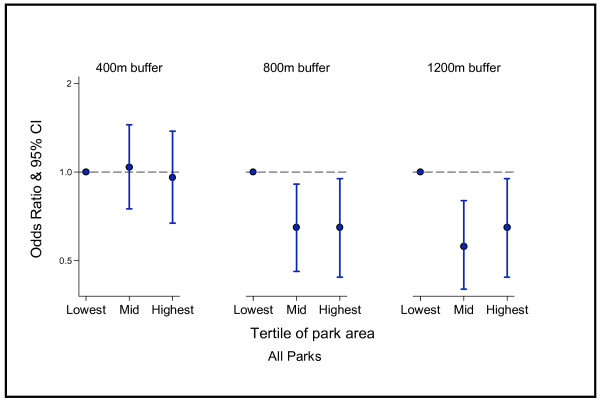
Park area of all parks: odds ratios for at least weekly walkers (all purposes) compared to less than weekly walkers.

As Figure 1 shows, there was no statistical evidence to support an association between park area for all parks and total walking frequency for the 400 m buffer. However, for the 800 m (OR 0.65, 95% CI 0.44-0.95) and 1200 m (OR 0.65, 95% CI 0.46-0.91) buffer, respondents with the highest amount of park area (highest park area tertile) were less likely to be at least weekly walkers compared to if they lived if in the lowest tertile for park area. Similarly for the mid tertile, at both the 800 m (OR 0.65, 95% CI 0.46-0.91) and 1200 m buffer (OR 0.56, 95% CI 0.4-0.8), people with access to the highest amount of park area were less likely to be at least weekly walkers.

### Large parks and walking

**Figure 2:**
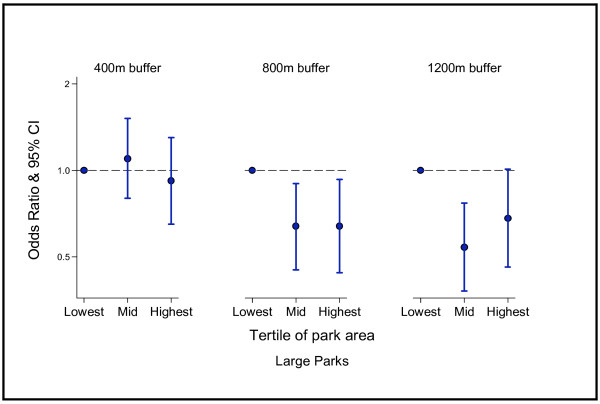
Park area of large parks: odds ratios for at least weekly walkers (all purposes) compared to less than weekly walkers.

Figure 2 presents the odds ratios for the effects of area of larger parks on total walking frequency. For parks within the 800 m (OR 0.64, CI 0.45-0.9) and 1200 m (OR 0.54, CI 0.38-0.77) buffer, people in the mid tertile for amount of ‘large park’ area in their neighbourhood were less likely to be at least weekly walkers, compared to if their neighbourhood had a lower amount of ‘large park’ area. A similar pattern of results arose at the highest tertile.

## Discussion

The results of this multilevel study provide important evidence that the relationship between park area and levels of walking is far from straightforward. Contrary to what we expected, respondents were less likely to walk on a weekly basis when they lived in areas with high amounts of parkland. These effects arose at both the 800 m and 1200 m buffers, but there were no significant findings at the 400 m buffer. Importantly, the size of parks appears to make no difference - results for ‘all parks’ and ‘larger parks’ were similar.

Of the studies looking specifically at the relationship between park access and walking, results have been mixed. Several studies have found positive associations between access and walking [29-31,35], others have found no significant relationship [32-34], negative associations [38], and mixed results [26,36]. Panter and Jones [34] reported results similar to ours when they found that there was a non-significant trend for those living closest to parks to be less likely to walk five or more times a week. Looking more broadly at the relationship between parks and physical activity or health generally (rather than walking), there are examples of similar counter intuitive [38,51,52], or non-significant negative relationships [21,34,53].

While the results are perplexing, there are a number of potential explanations. Firstly, it is highly possible that the types of neighbourhoods containing large areas of parkland differ from those offering less area of parkland. The results we observed may reflect area differences in urbanisation, street connectivity, population density, or land use mix. These are all aspects of the built environment that have previously been associated with walking levels [54-57]. If the areas in our study that contained high levels of parkland were also in outer suburbs with poor connectivity, few destinations and little diversity in land use mix, then we would expect results similar to those attained. Additionally, it is possible that those respondents with less park area in their neighbourhood may have had other destinations such as cafes, schools, shops and community facilities that they walked to. Such destinations, especially food shops and schools, are likely to be frequented by a higher proportion of the population, more frequently than recreational destinations. In support of this, Cerin and colleagues [58] found that residents in more commercial/industrial areas reported significantly more walking for transport than residents of areas with a recreational profile.

Secondly, we expected that the amount of park area would encourage walking by offering a destination for people to walk to and in, by improving the aesthetics of the neighbourhood, or alternatively by offering a short cut for people to walk through (compared to a street network journey). However, it is possible that parks are not regarded as a destination to walk to on a regular basis. Rather, it is possible that parks are seen as a place to kick the football in, play the sport prescribed by the space (i.e. lacrosse, if on a lacrosse field; football if on a football field), or have a picnic in. Furthermore, it is conceivable that path legibility has an over-riding impact on walking: people walking for transport may be more likely to seek direct routes through streets, and those walking recreationally/for exercise may seek clear/smooth pathways (i.e. not across ovals/grassland). In support of this, walking in the neighbourhood has been found to be influenced by footpaths, walking paths, local shops and perceived safety [59], as well as walking track length, having paths located closer to roads, and a greater number and variety of destinations [39]. Parks may in fact, offer less direct routes than the connectivity of an urban grid – they may be fenced or bounded by waterways or train lines.

Finally, our park dataset contained no information on qualitative aspects of parks such as the facilities offered by the different parks, or the perceived safety or aesthetics of the parks. Our dataset did not distinguish between parks with unkempt grass, parks with dense tree coverage, parks with manicured garden beds, and parks with adventure playgrounds. There is evidence that park usage is influenced by facilities/amenities [24,59], and aesthetics/attractiveness [30,59]. Evidence of qualitative differences in parks influencing usage also comes from the United Kingdom, where respondents closest to a formal park (with a structured path network and organised layout) were more likely to be sufficiently active, but other types of parks had no significant effect on being sufficiently active [17]. It is therefore possible that park quality may have varied across our sample, and influenced our results.

This study improves on previous studies in a number of ways. Firstly, most studies investigating park accessibility in terms of park area have either used neighbourhood level measures of park area (i.e. park area as a percentage of city acreage), or used a single buffer distance, (rather than multiple buffers). In this study, we used three different buffers specific to each respondent, and are therefore better able to understand at what distance park area may influence walking behaviour. Secondly, most previous studies have investigated the total amount of park area, which, depending on the source of geo-referenced parks, may include a vast number of small parks that may have little impact on activity levels. We use two measures of park area: total park area (all parks) as well as the area of larger parks in an attempt to understand whether the size of the park is important in encouraging walking. Thirdly, few studies have measured park area specific to each individual. Increasing sophistication of GIS technology is enabling increasingly complex analysis of neighbourhoods. By calculating buffer areas for each individual, we were able to investigate neighbourhood effects with much greater specificity than would otherwise be possible.

There are some limitations of this study. Firstly, as with all cross-sectional studies, any significant associations arising from the analysis cannot be interpreted as suggesting causation. Secondly, we did not control for the self-selection of people who perceive that there are benefits to physical activity, and select into an area that supports physical activity. If self-selection exists, but is not controlled for, there is the risk that the effect of the built environment on travel behaviour is estimated incorrectly. Importantly however, a recent paper examining the influence of self-selection on the relationship between park area and walking found that self-selection did not exclusively explain the relationship, and that those who placed greater importance on neighbourhood parks, were not more likely to live near more open space [60]. Thirdly, walking has been shown to vary by walking purpose [20]. It may be argued that the fact that we did not distinguish between walking purpose in our analysis may have led to imprecision or mis-estimation of the effect of park area on walking. In consideration of this, we tested for an interaction between park area and walking purpose, and found none. We also ran separate models for transport walking and recreational walking. However we found that our results held for both types of walking, and therefore chose to use total walking frequency as the outcome measure. Importantly too, and in defence of this general measure of walking, it is often difficult for both respondents (and analysts) to distinguish between walking trips on the basis of purpose. This may be particularly the case for parks, where it is conceivable that confusion may arise due to the ‘recreational’ nature of parks. If a person was to walk to the park with the intention of kicking the football, should they be classified as walking for recreation or transport?

A further limitation of the study is our measure of disability/injury, which did not distinguish between the type of injury or disability suffered by respondents, nor the extent to which it affected their daily life, in particular their mobility and capacity to walk. We ran separate exploratory analyses excluding respondents with a disability or injury. We found that the results did not change substantially other than an increase in the strength of the association between park area and walking for the 400 m radius, but that this remained non-significant (All Parks: p = 0.11 for tertile 1, p = 0.84 for tertile 2; Large Parks: p = 0.398 for tertile 1, p = 0.199 for tertile 2). As there was little effect on the results, we retained these respondents in the model, but included this variable as a confounder. Finally, our use of Euclidean buffers constitutes a potential limitation. While the use of Euclidean distance is considered more appropriate in determining open space access, it is possible that some pedestrians encounter network barriers en route to parks; Euclidean buffers may therefore overstate access.

## Conclusions

This study found that living in areas with moderate to high amounts of park area reduced the frequency of walking. Although these findings are counterintuitive, it is possible that they may be explained by other characteristics that may be negatively correlated with park area, such as connectivity and the presence of destinations. Importantly, while parks may not have an effect on walking levels, there are likely to be many other health benefits of having access to parks.

## Abbreviations

CCD: Census Collection District; LGA: Local Government Area; GIS: Geographic Information Systems; VicLANES: Victorian Lifestyle and Neighbourhood Environment Study; ARCUE: Australian Research Centre in Urban Ecology; ABS: Australian Bureau of Statistics; ASCO: Australian Standard Classification of Occupations.

## Competing interests

The authors declare that they have no competing interests.

## Authors' contributions

TK: study design, conceptualization of methods and analysis, wrote paper. AK: study design, contributed to drafts of paper. LT: contributed to conceptualization of methods and analysis. contributed to drafts of paper. RB: contributed to conceptualization of methods and analysis, contributed to drafts of paper. All authors read and approved the final manuscript.
